# Prenatal exposure to barium and arsenic and the odds of congenital heart defects in offspring: a nested case–control study within a birth cohort in Lanzhou, China

**DOI:** 10.3389/fpubh.2025.1597178

**Published:** 2025-05-01

**Authors:** Yun Dang, Jianhao Sun, Zhenzhen Wu, Baohong Mao, Qinglei Hang, Jie Huang, Xiaoli Zhao, Ji Xia, Cheng Chen, Wenxiang Yao, Dan Lu, Qing Liu

**Affiliations:** ^1^Gansu Provincial Maternity and Child-Care Hospital/Gansu Provincial Central Hospital, Lanzhou, China; ^2^Institute of Pathology, School of Basic Medical Sciences, Lanzhou University, Lanzhou, China; ^3^Northern Jiangsu People's Hospital Affiliated to Yangzhou University, Yangzhou, China; ^4^Institute of Translational Medicine, Medical College, Yangzhou University, Yangzhou, China; ^5^Department of Clinical Medicine, Medical College, Key Laboratory of Jiangsu Province University for Nucleic Acid & Cell Fate Manipulation, Yangzhou University, Yangzhou, China; ^6^Baiyin Second People's Hospital, Baiyin, China

**Keywords:** congenital heart defects, barium, arsenic, pregnancy, maternal blood, interaction

## Abstract

**Background:**

Previous studies have identified that exposure to heavy metals increases the prevalence of congenital heart defects (CHDs); however, limited information exists regarding the association between combined exposure to barium (Ba) and arsenic (As), and CHDs. This study aims to investigate the association between prenatal exposure to Ba and As (both independently and in combination) and the risk of CHDs in offspring.

**Methods:**

In a birth cohort study conducted in Lanzhou, China, a total of 97 mother-newborn pairs were designated as the case group, with an additional 194 pairs constituting the control group. The concentrations of Ba and As in maternal blood were quantified utilizing an inductively coupled plasma mass spectrometer. A multivariate logistic regression model was employed to examine the association between Ba and As exposure levels and the risk of neonatal CHDs and their subtypes. Interaction effects were further evaluated through the application of both additive and multiplicative models.

**Results:**

The concentration of As in the blood of pregnant women is positively correlated with Ba levels. Higher concentrations of maternal blood Ba level was associated with greater odds of CHDs (*p* = 0.008), including the isolated CHDs (*p* = 0.013), the multiple CHDs (*p* = 0.032), PDA (*p* = 0.014), and ASDs (*p* = 0.031); Similarly, higher concentrations of maternal blood As level was associated with greater odds of CHDs (*p* = 0.013), including the isolated CHDs (*p* = 0.016), the multiple CHDs (*p* = 0.003), PDA (*p* = 0.005), ASDs (*p* = 0.017), and AVSDs (*p* = 0.034). Elevated levels of barium and arsenic in maternal blood were significantly associated with increased odds of CHDs and their subtypes in offspring (All *p* < 0.05). Furthermore, a significant multiplicative interaction between Ba and As levels in maternal blood was identified in relation to total CHDs (*p* = 0.04).

**Conclusion:**

Exposure to Ba or As individually, as well as combined exposure to both, is significantly associated with an increased risk of CHDs in offspring.

## 1 Introduction

Congenital heart defects (CHDs) refer to congenital abnormalities involving the structure of the heart and large blood vessels and is the most common congenital disease in newborns, the worldwide prevalence of CHDs is 8 to 10‰ (1). Over the past 40 years, the total prevalence of CHDs at birth in China has continued to rise to 4.9‰ (2). CHDs is caused by genetic factors, environmental factors alone or both (3). The 3rd to 8th week of pregnancy is a critical period for the formation of the heart and large blood vessels, during which environmental factors have the greatest impact on the fetus, which is easy to cause fetal malformation (4). Previous studies have shown that many non-genetic factors such as maternal obesity, diabetes, systemic lupus erythematosus, and smoking may increase the risk of CHDs (5–8). Consequently, in contrast to genetic factors, environmental factors are more amenable to control and identification. By recognizing and mitigating environmental risk factors, the prevention of CHDs can be effectively pursued. Among the most critical environmental challenges globally is heavy metal pollution, which represents a significant threat to both environmental integrity and human health. Prolonged exposure to heavy metals has been associated with an increased risk of breast cancer, endometriosis, endometrial cancer, menstrual irregularities, spontaneous abortion, preterm delivery, and stillbirth (9). In addition, previous studies have found that exposure to heavy metals during pregnancy, including cadmium, mercury, lead, cobalt, titanium and stannum, is also a risk factor for CHDs (10–13).

Barium (Ba) is also a common heavy metal element, it occurs in nature only as the divalent cation Ba^2+^, mainly in sulfate and carbonates. In addition, Gansu Province’s ranking among the foremost regions in China for barium resource reserves (14). Barium salts are used in many products, such as plastics, ceramics, adhesives, drilling fluids, paints and hair dyes (15, 16). High levels of barium exposure can occur in some occupations, while environmental barium exposure can be low-level and chronic, with non-occupational human exposure primarily through water, food, and soil (17). Previous studies have shown that barium can be detected in the aorta, brain, eyes, skeletal muscle, spleen, pancreas, placenta, hair, blood, urine, and feces (17). The most important toxic effects of chronic barium exposure through oral or inhalation include pulmonary edema, gastrointestinal bleeding, heart failure, kidney failure, and respiratory failure (16). Animal studies on the reproductive and developmental effects of barium exposure indicate that high levels may result in abnormal ovarian morphology, spontaneous abortion, restricted fetal growth, and fetal death (17, 18). The relationship between barium exposure and CHDs remains inconclusive. A multi-center case–control study conducted by Zhang in China revealed that the median concentration of barium in the hair of pregnant women with CHDs-affected offspring was significantly higher compared to that of the control group (19). Conversely, a case–control study by Zierler in the United States found no significant association between barium levels in drinking water consumed during pregnancy and the overall prevalence of CHDs in offspring (20).

Arsenic (As) is a naturally occurring metalloid with toxic properties that is widely distributed in the environment, including in soil, water, and living organisms. It exists in various forms, such as trivalent and pentavalent states, as well as inorganic and organic compounds, with trivalent arsenic being the most toxic form (21). Arsenic contamination represents a significant global public health concern, predominantly attributed to anthropogenic activities including the extraction and processing of arsenic-bearing minerals, the widespread application of arsenic-based compounds, and the combustion of coal (22, 23). The human body is primarily exposed to arsenic through three main routes: ingestion via the digestive tract, inhalation through the respiratory system, and dermal contact in occupational settings (24, 25). Arsenic enters the body and primarily accumulates in the liver, kidneys, lungs, bones, skin, hair, nails, and other tissues and organs, posing significant risks to human health. Numerous studies have confirmed that environmental arsenic exposure is a significant risk factor for various cancers, including those of the lung, bladder, liver, kidney, and non-melanoma skin (24, 26). In addition, arsenic is capable of crossing the placental barrier, resulting in dual toxic effects on both the fetus and the mother (27). A national cohort study conducted in Denmark demonstrated that maternal exposure to low concentrations of arsenic in drinking water (0.5–0.9 μg/L) is associated with an increased risk of CHDs in offspring (28). In a similar vein, a study conducted in China revealed that increased arsenic levels in the hair of pregnant women were associated with a higher risk of coronary heart disease in their offspring (29).

Currently, research on the association between barium exposure during pregnancy and cardiac development in offspring remains inconclusive. Furthermore, the potential effects of combined exposure to barium and arsenic during pregnancy on CHDs in offspring have not been investigated. To investigate the relationship between maternal barium and arsenic exposure alone and combined exposure to maternal CHDs risk, we conducted a prospective nested case–control study in Lanzhou, China.

## 2 Methods

### 2.1 Study population

A birth cohort study was carried out between 2010 and 2012 at the Gansu Provincial Maternity and Child Care Hospital in Lanzhou, China (30). In our previous research, further details regarding the recruitment procedure were provided (12). A total of 10,542 people in this birth cohort completed baseline questionnaires and blood samples. According to the exclusion criteria, the study excluded 310 cases of multiple births, 53 cases of stillbirths, 88 cases involving non-CHDS birth defects, and 1 case with incomplete information. Based on the diagnosis of CHDs in singleton births, a total of 97 mother-newborn pairs were included as the case group; according to the assessment of singleton births as healthy, full-term, and with normal birth weight, and meeting the matching conditions of maternal age within 2 years of the case group and residing in the same neighborhood, a total of 194 mother-newborn pairs were included as the control group at a 1: 2 ratio. Ultimately, a total of 291 mother-newborn pairs were included as the study subjects. This research project was conducted with the informed consent of pregnant women and with the approval of the Medical Ethics Committee of Gansu Provincial Maternal and Child Health Hospital.

### 2.2 Questionnaire interview and sample collection

After pregnant women participating in the survey signed the informed consent form, trained investigators completed the filling out of the “China Birth Cohort Study – Maternal Health Survey Questionnaire,” jointly designed by Gansu Provincial Maternal and Child Health Hospital and the Yale School of Public Health, through face-to-face interviews. Finally, we integrated the following variables into our analysis of the study population: maternal age, maternal education, age of menarche, pregnancy history, childbearing history, health education before pregnancy, morning sickness, premature birth, delivery mode, and newborn birth weight.

Blood samples were collected from pregnant women upon hospital admission prior to delivery. In the morning, 3 mL of median cubital venous blood was drawn from fasting participants, along with 7 mL collected in specialized vacuum tubes containing EDTA anticoagulant. After collection, the samples were gently inverted to ensure proper mixing and stored at −80°C. All sample details were recorded in an electronic biological sample database.

### 2.3 Diagnosis and classification of CHDs

Fetal echocardiography is a widely accessible and commonly utilized diagnostic tool for detecting CHDs (31). Senior sonographers with extensive experience conducted the examinations in accordance with the guidelines and standards set by the American Society of Echocardiography and the International Society of Ultrasound in Obstetrics and Gynecology (32, 33). Diagnostic imaging was performed using multiple views, with CHDs diagnoses based on grayscale imaging, color Doppler, and pulsed-wave Doppler assessments.

Each type of CHDs is categorized based on its severity as “isolated,” “multiple,” or “syndromic.” Additionally, we classified these anomalies using clinical phenotypes and the International Classification of Diseases, 10th Revision (ICD-10) codes (Q20–Q28). Further details regarding the classification of CHDs are available in our previous research (12).

### 2.4 Blood samples analyzed for barium and arsenic

The frozen blood samples were thawed at ambient temperature, and precisely 200 μL of each sample was transferred into a polytetrafluoroethylene digestion vessel. Subsequently, 1 mL of 68% HNO₃ and 200 μL of 40% H₂O₂ were added sequentially. After ensuring that all bubbles had completely dissipated within the digestion vessel, the lid and steel cap were securely sealed. The sealed vessels were then placed in an oven and maintained at 175°C for 6 to 8 h. Upon cooling, the digestate was transferred to a 10 mL centrifuge tube and diluted to a final volume of 10 mL with deionized water, followed by thorough mixing prior to instrumental analysis. A blank solution was prepared by treating 200 μL of deionized water using the same procedure as the blood samples.

Subsequently, considering the widespread application of inductively coupled plasma mass spectrometry (ICP-MS) in environmental heavy metal detection (34), the concentrations of barium and arsenic in the blood of pregnant women were analyzed using ICP-MS (ICAP RQ; Thermo Fisher, United States). After tuning the ICP-MS instrument to optimal conditions, Sc^45^, Ge^72^, Rh^103^, and Re^187^ were selected as internal standards to mitigate matrix effects arising from non-spectrometric interferences. Calibration curves for barium and arsenic were then established, yielding correlation coefficients (R^2^) of 0.9999 and 1.0000, respectively. Both R^2^ values exceeded 0.9990, demonstrating excellent linearity within the tested concentration ranges and confirming the suitability of the method for sample analysis. Next, the blank solution was analyzed 11 consecutive times. The standard deviation (SD) of the ratio of the barium and arsenic counts per second (CPS) to the internal standard CPS was determined. The limits of detection (LOD) were calculated as three times the SD divided by the slope of the respective calibration curve (35). The LOD for barium and arsenic in whole blood are 0.206 and 0.044 μg/L, respectively. Concentrations below the LOD are recorded as zero.

### 2.5 Statistical analysis

Data were processed and analyzed using the SPSS 25.0 statistical software (IBM, Chicago, United States), with categorical variables expressed as percentages (%). Quantitative data that conform to a normal distribution are represented as the mean ± SD, while quantitative data that do not conform to a normal distribution are expressed using interquartile ranges. Univariate analysis between the case and control groups involved the use of chi-square tests for categorical variables, and for continuous variables that conform to a normal distribution, t-tests were employed; for those that do not conform to a normal distribution, rank sum tests were used. We further examined the distribution patterns of barium and arsenic concentrations in the study subjects and evaluated the inter-element correlation between these two trace elements.

In the absence of established reference ranges for barium and arsenic levels in the blood of pregnant women, this study utilized methods from previous research to determine optimal cut-off values. These values were calculated by constructing a receiver operating characteristic (ROC) curve (36, 37). The optimal cut-off values for barium and arsenic concentrations in maternal blood were determined to be 213.84 and 7.28 μg/L, respectively. Based on these cut-off values, the concentrations of barium and arsenic in the blood of pregnant women were categorized into low exposure and high exposure groups. To examine the relationship between prenatal exposure to barium and arsenic and the risk of coronary heart disease in offspring, odds ratios (ORs) and 95% confidence intervals (CIs) were calculated. The analysis used exposure concentrations below the optimal cut-off value as the reference point. A *p*-value of less than 0.05 indicated statistically significant differences, and factors with *p*-values less than 0.05 from the univariate analysis were included in the multivariate analysis. We employed both multiplicative and additive interaction models to examine the modifying effects of barium and arsenic on CHDs.

## 3 Results

### 3.1 Characteristics of participants

The 97 cases of CHDs comprised isolated CHDs (43 cases), multiple CHDs (48 cases), syndromic CHDs (6 cases), patent ductus arteriosus (PDA, 70 cases), atrial septal defects (ASDs, 48 cases), ventricular septal defects (VSDs, 7 cases), and atrioventricular septal defects (AVSDs, 4 cases). Table 1 presents the baseline characteristics of the study subjects. Notably, significant differences were observed between the two groups in premature birth, delivery mode, newborn birth weight, barium levels in maternal blood, and arsenic levels in maternal blood (*p* < 0.05), while no significant differences were found in other characteristics. The correlation analysis revealed a positive association between the arsenic concentration in the blood of pregnant women and the concentration of barium (*r* = 0.863, *p* < 0.001).

**Table 1:** Study sample characteristics.

Variable	Total	Cases	Control	*P*-value^#^
	*N* = 291 (%)	*N* = 97 (%)	*N* = 194 (%)	
Maternal age (years, *n*)				0.082
≤35	254 (87.3)	80 (82.5)	174 (89.7)	
>35	37 (12.7)	17 (17.5)	20 (10.3)	
Maternal education				0.658
Junior high or below	94 (32.3)	34 (35.1)	60 (30.9)	
Technical secondary school, high school	45 (15.5)	16 (16.5)	29 (14.9)	
Undergraduate college or above	152 (52.2)	47 (48.4)	105 (54.2)	
Age of menarche (years, *n*)				0.373
≤14	199 (68.4)	63 (64.9)	136 (70.1)	
>14	92 (31.6)	34 (35.1)	58 (29.9)	
Pregnancy history				0.863
Yes	106 (36.4)	36 (37.1)	70 (36.1)	
No	185 (63.6)	61 (62.9)	124 (63.9)	
Childbearing history				0.791
Yes	96 (33.0)	31 (32.0)	65 (33.5)	
No	195 (67.0)	66 (68.0)	129 (66.5)	
Health education before pregnancy				0.559
Yes	128 (44.0)	45 (46.4)	83 (42.8)	
No	163 (56.0)	52 (53.6)	111 (57.2)	
Morning sickness				0.618
Yes	156 (53.6)	50 (51.5)	106 (54.6)	
No	135 (46.4)	47 (48.5)	88 (45.4)	
Premature birth				<0.001^*^
Yes	29 (10.0)	29 (29.9)	0 (0.0)	
No	262 (90.0)	68 (70.1)	194 (100.0)	
Delivery mode				<0.001^*^
Vaginal delivery	169 (58.1)	41 (42.3)	128 (66.0)	
Cesarean delivery	122 (41.9)	56 (57.7)	66 (34.0)	
Newborn birth weight (g)				<0.001^*^
<2,500	27 (9.3)	27 (27.8)	0 (0.0)	
2,500-	253 (86.9)	59 (60.8)	194 (100.0)	
≥4,000	11 (3.8)	11 (11.4)	0 (0.0)	
Barium levels in maternal blood				<0.001^*^
Low concentration (<213.84 μg/L)	243 (83.5)	175 (90.2)	68 (70.1)	
High concentration (≥213.84 μg/L)	48 (16.5)	19 (9.8)	29 (29.9)	
Arsenic levels in maternal blood				<0.001^*^
Low concentration (<7.28 μg/L)	249 (85.6)	178 (91.8)	71 (73.2)	
High concentration (≥7.28 μg/L)	42 (14.4)	16 (8.2)	26 (26.8)	

#*p*-values were derived using the chi-square test.*Significant results, *p*-value < 0.05.

### 3.2 The association between maternal blood barium concentrations and the prevalence of CHDs in offspring

Maternal blood samples were stratified into categories of low and high barium concentrations based on predetermined cut-off values to facilitate a detailed analysis of the association between varying barium levels and the odds of CHDs in offspring. As shown in Table 2, the aOR^2^ model reveals an increased odds of CHDs (aOR^2^ = 2.750, 95% CI [1.309–5.781]), including the isolated CHDs (aOR^2^ = 3.191, 95% CI [1.283–7.936]), the multiple CHDs (aOR^2^ = 2.719, 95% CI [1.091–6.777]), PDA (aOR^2^ = 2.754, 95% CI [1.229–6.170]), and ASDs (aOR^2^ = 2.723, 95% CI [1.096–6.768]), in the group with high maternal blood barium concentrations. These findings suggest that maternal blood barium exposure may elevate the odds of CHDs in offspring.

**Table 2:** Risks for fetal CHDs in different barium in maternal blood concentrations.

Group	Levels	Cases	Controls	aOR^1^ (95% CI)	aOR^2^ (95% CI)
All CHDs	Low	68	175	Reference	Reference
	High	29	19	4.261 (2.212–8.206)	2.750 (1.309–5.781)
The isolated CHDs	Low	29	175	Reference	Reference
	High	14	19	5.027 (2.217–11.399)	3.191 (1.283–7.936)
The multiple CHDs	Low	35	175	Reference	Reference
	High	13	19	3.825 (1.674–8.742)	2.719 (1.091–6.777)
The Syndrome CHDs	Low	4	175	Reference	Reference
	High	2	19	6.366 (0.974–41.599)	2.761 (0.283–26.988)
PDA	Low	49	175	Reference	Reference
	High	21	19	4.446 (2.169–9.113)	2.754 (1.229–6.170)
ASDs	Low	36	175	Reference	Reference
	High	12	19	3.463 (1.503–7.977)	2.723 (1.096–6.768)
VSDs	Low	4	175	Reference	Reference
	High	3	19	6.976 (1.392–34.957)	4.136 (0.706–24.220)
AVSDs	Low	3	175	Reference	Reference
	High	1	19	3.262 (0.318–33.418)	0.960 (0.058–15.833)

Logistic regression was used to calculate odds ratios and 95% CIs; Based on the optimal cut-off value derived from the ROC curve analysis, barium concentrations in the blood of pregnant women can be categorized into high and low concentration groups. The aOR^1^ models are calibrated for, premature birth, delivery mode, and newborn birth weight. The aOR^2^ model further corrects the arsenic concentration based on the aOR^1^ model.

aOR, adjusted odds ratio; CHDs, congenital heart defects; PDA, patent ductus arteriosus; ASDs, atrial septal defects; VSDs, ventricular septal defects; AVSDs, atrioventricular septal defects.

### 3.3 The association between maternal blood arsenic concentrations and the prevalence of CHDs in offspring

Maternal blood samples were stratified into categories of low and high arsenic concentrations based on predetermined cut-off values to facilitate a detailed analysis of the association between varying arsenic levels and the odds of CHDs in offspring. As shown in Table 3, the aOR^2^ model reveals an increased odds of CHDs (aOR^2^ = 2.793, 95% CI [1.247–6.254]), including the isolated CHDs (aOR^2^ = 3.513, 95% CI [1.263–9.775]), the multiple CHDs (aOR^2^ = 3.830, 95% CI [1.600–9.166]), PDA (aOR^2^ = 3.390, 95% CI [1.438–7.992]), ASDs (aOR^2^ = 2.983, 95% CI [1.220–7.294]), and AVSDs (aOR^2^ = 27.805, 95% CI [1.287–600.889]), in the group with high maternal blood arsenic concentrations. These findings suggest that maternal blood arsenic exposure may elevate the odds of CHDs in offspring.

**Table 3:** Risks for fetal CHDs in different arsenic in maternal blood concentrations.

Group	Levels	Cases	Controls	aOR^1^ (95% CI)	aOR^2^ (95% CI)
All CHDs	Low	71	178	Reference	Reference
	High	26	16	4.621 (2.272–9.402)	2.793 (1.247–6.254)
The isolated CHDs	Low	31	178	Reference	Reference
	High	12	16	5.928 (2.363–14.873)	3.513 (1.263–9.775)
The multiple CHDs	Low	36	178	Reference	Reference
	High	12	16	3.708 (1.617–8.502)	3.830 (1.600–9.166)
The Syndrome CHDs	Low	4	178	Reference	Reference
	High	2	16	14.691 (1.644–131.321)	8.283 (0.683–100.505)
PDA	Low	50	178	Reference	Reference
	High	20	16	5.339 (2.470–11.544)	3.390 (1.438–7.992)
ASDs	Low	38	178	Reference	Reference
	High	10	16	2.928 (1.234–6.948)	2.983 (1.220–7.294)
VSDs	Low	4	178	Reference	Reference
	High	3	16	8.269 (1.511–45.272)	4.902 (0.79–30.186)
AVSDs	Low	2	178	Reference	Reference
	High	2	16	27.246 (1.782–416.484)	27.805 (1.287–600.889)

Logistic regression was used to calculate odds ratios and 95% CIs; Based on the optimal cut-off value derived from the ROC curve analysis, arsenic concentrations in the blood of pregnant women can be categorized into high and low concentration groups. The aOR^1^ models are calibrated for premature birth, delivery mode, and newborn birth weight. The aOR^2^ model further corrects the barium concentration based on the aOR^1^ model.

aOR, adjusted odds ratio; CHDs, congenital heart defects; PDA, patent ductus arteriosus; ASDs, atrial septal defects; VSDs, ventricular septal defects; AVSDs, atrioventricular septal defects.

### 3.4 Interaction effects between barium and arsenic

As shown in Table 4, the combined high levels of barium and arsenic in maternal blood were significantly associated with an increased odds of CHDs in offspring. The joint high levels of barium and arsenic significantly elevated the odds of total CHDs (aOR = 12.35, 95% CI [4.39–34.74]), the isolated CHDs (aOR = 16.47, 95% CI [4.98–54.46]), the multiple CHDs (aOR = 9.89, 95% CI [3.00–32.64]), and the syndromic CHDs (aOR = 28.7, 95% CI [3.33–247.75]). We also observed that the combined high levels of barium and arsenic were significantly associated with an increased odds of PDA (aOR = 13.53, 95% CI [4.54–40.3]), ASDs (aOR = 8.85, 95% CI [2.61–29.99]), VSDs (aOR = 29.29, 95% CI [4.45–192.71]), and AVSDs (aOR = 23.33, 95% CI [1.31–415.89]). A multiplicative interaction between barium and arsenic levels in maternal blood was observed in relation to total CHDs (aOR = 6.04, 95% CI [1.07–34.11]). However, no significant additive interaction was detected. Similarly, neither multiplicative nor additive interactions were found in analyses of specific CHDs subtypes.

**Table 4:** Analyzes the effect of barium and arsenic interaction in maternal blood on offspring CHDs using multivariate logistic regression.

Exposure	All CHDs		The isolated CHDs		The multiple CHDs		The Syndrome CHDs	
	Case/control (*n*)	aOR (95% CI)	Case/control (*n*)	aOR (95% CI)	Case/control (n)	aOR (95% CI)	Case/control (*n*)	aOR (95% CI)
Low Ba and low As	63/164	Reference	27/164	Reference	32/164	Reference	4/164	Reference
Low Ba and high As	5/11	1.29 (0.42–4.01)	2/11	1.42 (0.28–7.19)	3/11	1.31 (0.33–5.18)	0/11	–
High Ba and low As	8/14	1.58 (0.62–4.01)	4/14	1.70 (0.50–5.80)	4/14	1.62 (0.49–5.37)	0/14	–
High Ba and high As	21/5	12.35 (4.39–34.74)	10/5	16.47 (4.98–54.46)	9/5	9.89 (3.00–32.64)	2/5	28.7 (3.33–247.75)
Multiplicative interaction		6.04 (1.07–34.11)		6.84 (0.73–64.41)		4.65 (0.57–38.06)		–
RERI		10.48 (−2.19–23.15)		14.35 (−5.03–33.73)		7.95 (−3.77–19.67)		29.7 (−32.16–91.57)
AP		0.85 (0.63–1.07)		0.87 (0.64–1.10)		0.80 (0.47–1.14)		1.03 (0.96–1.11)
SI		12.97 (0.98–172.22)		13.84 (0.72–266.19)		9.51 (0.44–206.8)		–

Exposure	PDA		ASDs		VSDs		AVSDs	
	Case/control (n)	aOR (95% CI)	Case/control (n)	aOR (95% CI)	Case/control (n)	aOR (95% CI)	Case/control (n)	aOR (95% CI)
Low Ba and low As	44/164	Reference	34/164	Reference	4/164	Reference	2/164	Reference
Low Ba and high As	5/11	1.92 (0.61–6.09)	2/11	0.80 (0.17–3.91)	0/11	–	1/11	17.43 (0.69–437.67)
High Ba and low As	6/14	1.76 (0.63–4.94)	4/14	1.51 (0.46–5.02)	0/14	–	0/14	–
High Ba and high As	15/5	13.53 (4.54–40.3)	8/5	8.85 (2.61–29.99)	3/5	29.29 (4.45–192.71)	1/5	23.33 (1.31–415.89)
Multiplicative interaction		4.00 (0.65–24.54)		7.27 (0.75–70.36)		–		–
RERI		10.85 (−3.76–25.46)		7.53 (−3.22–18.28)		30.29 (−24.89–85.47)		6.91 (−59.11–72.93)
AP		0.80 (0.52–1.09)		0.85 (0.55–1.15)		1.03 (0.97–1.10)		0.30 (−2.07–2.67)
SI		7.46 (1.02–54.33)		24.61 (0.02–32950.47)		–		1.45 (0.04–51.07)

Logistic regression was used to calculate odds ratios and 95% CIs; The aOR models are calibrated for premature birth, delivery mode, and newborn birth weight.

aOR, adjusted odds ratio; Ba, barium; As, arsenic; CHDs, congenital heart defects; PDA, patent ductus arteriosus; ASDs, atrial septal defects; VSDs, ventricular septal defects; AVSDs, atrioventricular septal defects.

## 4 Discussion

The objective of this study was to examine the potential interaction between maternal exposure to barium and arsenic during pregnancy and the associated risk of CHDs in offspring. Utilizing multifactorial logistic regression analysis, we identified a significant association between individual exposures to barium and arsenic and an increased risk of CHDs in offspring. Our findings indicate that pregnant women with elevated blood concentrations of barium and arsenic are at a significantly heightened risk of having offspring with CHDs compared to those with lower levels of these substances. Additionally, a significant multiplicative interaction between maternal blood levels of barium and arsenic was observed concerning the overall incidence of CHDs.

While a consensus on the relationship between barium exposure and CHDs has yet to be reached, our study’s findings align with those of Zhang’s multi-center case–control study conducted in China (19). Specifically, we observed that elevated barium levels in the blood of pregnant women are associated with an increased risk of CHDs (aOR^2^ = 2.750, 95% CI [1.309–5.781]; *p* = 0.008). Furthermore, our study indicates that the degree of barium exposure in pregnant women’s blood correlates with the risk of some CHDs subtypes. Although the mechanism underlying barium toxicity is not yet fully understood, it is believed to involve barium’s role as a competitive antagonist of K^+^ channels, which disrupts the passive efflux of intracellular K^+^. Additionally, barium interferes with the metabolic regulation of K^+^ levels (38). In addition, an animal study found that barium causes heart damage, disrupts REDOX balance, impairs cholinergic function, and inhibits the activity of Na^+^-K^+^ ATPase and Mg^2+^ ATPase. However, the combination of selenium and vitamin C significantly enhanced the antioxidant defense system in barium-treated rats. The mechanisms underlying this effect include the scavenging of free radicals and the improvement of antioxidant status (39).

Previous epidemiological studies have demonstrated an association between maternal arsenic exposure and an increased risk of CHDs in offspring, primarily through the measurement of arsenic concentrations in maternal drinking water and hair (28, 29, 40, 41). Consistent with these findings, our case–control studies assessing arsenic levels in the blood of pregnant women also identified a similar association with CHDs in their offspring. Animal studies have also shown that offspring of mice exposed to arsenic-contaminated drinking water before and during pregnancy exhibit cardiovascular abnormalities, including significantly elevated rates of atrial septal defects and other heart malformations (42). Arsenic exposure may contribute to heart disease through several mechanisms during cardiac development. Firstly, research has demonstrated that arsenic has the potential to disrupt epithelial-mesenchymal transformation during cardiac development by interfering with the TGF-β/Smad signaling pathway (43). Secondly, a study conducted on animals indicates that folic acid supplementation during the periconception period may mitigate the cardiotoxic effects of arsenic. This protective mechanism is hypothesized to involve a reduction in the acetylation levels of histone H3K9 within cardiac tissues, which subsequently leads to decreased expression levels of Mef2C, a factor that may contribute to heart development in fetal rats (44).

Previous research has not thoroughly investigated the interaction between barium and arsenic in relation to CHDs. Our study demonstrated that pregnant women with elevated blood levels of barium and arsenic face a significantly higher risk of having offspring with CHDs compared to those with lower levels of these substances. Additionally, we identified a significant multiplicative interaction between maternal blood levels of barium and arsenic and the occurrence of total CHDs. The study found that exposure to barium or arsenic in pregnant women elevated biomarkers of oxidative stress (45). These metals may induce oxidative stress through various mechanisms, such as binding to protein sulfides and directly disrupting the antioxidant system by depleting glutathione (46, 47), ultimately contributing to adverse pregnancy outcomes. However, the molecular mechanisms underlying these interactions require further investigation.

This study employed a nested case–control design within a prospective maternal and offspring birth cohort of 10,542 individuals who completed baseline questionnaires and provided blood samples, establishing a robust foundation for our research. However, there are some limitations to consider. First, in the analysis of CHDs subtypes, the sample size within each subgroup decreases as stratification levels increase, potentially impacting statistical power. This may explain the absence of multiplicative or additive interactions in the CHDs subtype analysis. Second, we only measured the concentrations of arsenic and barium during the third trimester prior to hospital delivery and could not assess changes in arsenic and barium levels during pregnancy. Third, while this study examined the association between barium and arsenic exposure during pregnancy and CHDs in offspring from a population-based perspective, further research is needed to investigate the metabolic dynamics and pathogenesis of CHDs.

## 5 Conclusion

Overall, our study demonstrates that exposure to barium or arsenic individually, as well as combined exposure to both, is significantly associated with an increased risk of CHDs in offspring. Additionally, a significant multiplicative interaction was observed between maternal blood levels of barium and arsenic and the occurrence of total CHDs. Further research is required to investigate the mechanisms through which barium and arsenic exposure during pregnancy contribute to the development of CHDs.

## Data Availability

The raw data supporting the conclusions of this article will be made available by the authors, without undue reservation. Inquiries for access to the datasets should be directed to 1038817191@qq.com.
